# Mental Treatment

**Published:** 1910-08-13

**Authors:** Frederick Dixon


*To the Editor of* The Hospital.

### MENTAL TREATMENT.

*To the Editor of* The Hospital.

Sir,—Will you, as a simple matter of fairness, allow me to allude to a passage in the article on mental healing in your issue of July 30, in which a statement is made with respect to Christian Science which is as exactly the reverse of the truth as anything could be? "In the case of Christian Science," the writer says, "the practitioners demand that the patient shall have faith in a supernatural origin for the power which flows from them." This statement was no doubt made in perfectly good faith, but it reverses what Christian Science does teach as completely as could be possible.

First of all, Christian Scientists deny utterly that there is anything supernatural in Christian Science healing; they insist, on the contrary, that, in the words of Mrs. Eddy, on p. 591 of "Science and Health," what the world terms a miracle is "that which is divinely natural, but must be learned humanly." Secondly, no Christian Science workers have ever maintained that any healing power ever flowed from them. What they do maintain is that healing in Christian Science is brought about through a knowledge of spiritual causation, and that it was this to which Jesus referred when he said, "Ye shall know the truth, and the truth shall make you free." Finally, it has never been said that, in order to be healed by Christian Science, patients must have faith in it. Mrs. Eddy, indeed, says precisely the reverse on p. 358 of "Science and Health," where, answering the criticism that Christian Scientists heal through arousing the belief that they have a wonderful power, she asks whether Church members would not have a greater faith in their own clergymen, and yet these clergymen would admit that they could not cure their friends by their faith in them. I think you will admit that, whatever anybody may think of Christian Science healing, it would have been difficult to misstate it more completely than in the sentence I have quoted from your paper.

As a matter of fact, healing in Christian Science is so intensely simple that it is difficult to get people who do not understand Christian Science to recognise how simple it is. It is brought about by a recognition of the fact that all sickness is a subjective condition of the human mind, and that just exactly in proportion as anyone grasps the fact that this human mind can be governed by Truth, he begins to acquire, in no matter how limited a degree, the Mind that was in Christ Jesus. Again, exactly in the proportion in which he lets this Mind be in him, he obliterates the impressions of a human mind, tortured with a belief in the power and reality of evil. As this change takes place, there necessarily comes a change in the subjective condition, for, as Mrs. Eddy writes on p. 312 of "Science and Health," "whatever is learned through material sense must be lost because such so-called knowledge is reversed by the spiritual facts of being in Science. That which material sense calls intangible is found to be substance. What to material sense seems substance becomes nothingness as the sense-dream vanishes and reality appears."—Yours truly, Frederick Dixon.

[We have inserted this letter, but it is manifestly impossible to throw our columns open for a discussion on the subject of "Christian" Science.—Ed. The Hospital.]

